# Sex, drugs and high fat diet: Characterizing HFpEF in female C57BL6/J mice

**DOI:** 10.1016/j.jmccpl.2024.100063

**Published:** 2024-01-23

**Authors:** Joshua Travers, Emma L. Robinson

**Affiliations:** Department of Medicine, Division of Cardiology, University of Colorado Anschutz Medical Campus, Aurora, CO, USA; Consortium for Fibrosis Research & Translation, University of Colorado Anschutz Medical Campus, Aurora, CO, USA

Whilst we often think of cardiovascular disease as a modern affliction, strongly incited by risk factors commonplace in our population, the earliest reports of cardiovascular disease, including atherosclerosis and myocardial infarction, date back to the ancient Egyptians and Greeks. It was in 1933 that the first modern concept of heart failure (HF) was coined by Thomas Lewis: “a condition in which the heart fails to discharge its contents adequately.” Since, the definitions of HF have remained centered around this concept of an end-stage pathology of the heart, leading to the heart unable to pump sufficient blood to the head and body to maintain good health. HF poses a major societal burden, with a 5-year mortality rate of greater then 50 % and >64 million people affected globally [1]. Clinical guidelines are now published by the European Society of Cardiology and the American Heart Association/American College of Cardiology every few years concerning the diagnosis and treatment approaches for acute and chronic HF.

Historically, as stated by Thomas Lewis, HF was understood to be the inability of the heart to pump effectively with patients presenting with a reduced ejection fraction. Patients are diagnosed with HF with reduced ejection fraction (HFrEF) if they present with a left ventricular EF of <40 %, upon echocardiographic assessment, along with other diagnostic parameters such as elevated circulating natriuretic peptides and signs and symptoms such as dyspnea and fatigue.

However, more recent advances in *in vivo* imaging as well as comprehensive cohort studies have revealed that up to half of clinical HF patients present with remarkably similar signs and symptoms to the classic HFrEF but have preserved ejection fraction [2]. Instead, this 50 % of the HF patient population have compromised diastolic function – the heart being unable to sufficiently relax in diastole due to ventricular stiffening. Whilst clinical outcomes are reportedly similar between HFrEF and HFpEF patients, there exist demographic differences in incidence. HFpEF patients are older, predominantly female and are more likely to present with comorbidities, such as hypertension, obesity, diabetes, atrial fibrillation and renal disease [3,4], all of which are associated with worse outcomes.

Only in 2022 was the first and only pharmacological drug, the SGLT2 inhibitor empagliflozin, approved by the FDA for HF patients with preserved ejection fraction [5]. Our toolbox for treating those HF patients presenting with diastolic dysfunction, remains very thin on the ground. The basis for drug target discovery and pharmaceutical development is an adequate understanding of the molecular basis of disease. In order to increase knowledge for its mechanistic underpinnings, appropriate laboratory and pre-clinical models of HFpEF are required. Clinically relevant, reproducible and validated pre-clinical models of this complex, multi-factorial syndrome remain an area for improvement and progress in translational cardiovascular research.

The earliest small animal models of HFpEF focused on producing concentric LV hypertrophy prior to decompensation through surgical interventions such as transaortic constriction. However, whilst ejection fraction was still ‘normal’ at end point using these models, they failed to encapsulate progressive comorbidity-driven ventricular and vascular stiffness, endothelial cell dysfunction, cardiac fibrosis, impaired myocardial relaxation and pulmonary edema. One pressure overload model that is exempt from these shortcomings is that of loose aortic banding in juvenile male felines, which does induce stable cardiopulmonary compromise and diastolic dysfunction [6]. In addition, the Obese ZSF1 rat model of cardiometabolic syndrome exhibits many features of human disease including diastolic dysfunction, hypertension, nephropathy and diabetes, and responds positively to SGLT2 inhibition [7]. However, performing mechanistic investigative studies in larger animals lacks economic viability, have high administrative demands and can pose ethical concerns.

In 2019, the lab of Joseph Hill published one of the first multi-comorbidity driven murine models of HFpEF in Nature [8]. In this model, male C57BL/6N mice are fed a high fat diet (HFD; 60 % calories from lard) and systemic administration of N(ω)-nitro-L-arginine methyl ester (L-NAME), inducible NO synthase (iNOS) inhibitor, to progressively induce obesity, insulin resistance and hypertension. By 15 weeks, the mice present with LV hypertrophy and increased LV filling pressure and strain with a preserved LVEF of >80 %. Other indicators of pathology that reflect the clinical phenotype include pulmonary congestion, cardiac fibrosis, increased vascular stiffness, myocardial capillary rarefaction and compromised exercise intolerance. This phenotype was stable and sustained for up to one year in this ‘two-hit’ comorbidity model of HFpEF.

A follow-up article from the same group in the same year confirmed that the same dual hit model does not fully phenocopy in female C57BL6/N mice of the same age [9]. This was determined to be independent of the cardioprotective role of estrogen, with even ovariectomized female mice being resistant to development of HFpEF pathophysiology and symptoms.

In this issue of JMCC Plus, Srinivas et al. demonstrate that a near-identical ‘two-hit’ model, HFD plus L-NAME for 5 or 16 weeks, does result in a HFpEF-like phenotype in female animals [10]. Five weeks of HFD, (60 % calories from fat) and L-NAME (0.5 g/L in the drinking water) in young female mice incited a significant increase in E/E' ratio, indicative of compromised diastolic function and elevated left ventricular mass with preserved ejection fraction ≥65 %. A significant increase in wet lung mass indicates congestion, reduced glucose tolerance, increased circulating BNP, a reduction in the measured glomerular filtration rate and some elevation in circulating markers that additionally suggest kidney disease are reported. All such features are sustained or worsened by 16 weeks of the model, with an increase in qualitative cardiac fibrosis and blood BNP levels exceeding the 125 pg/mL threshold supporting a diagnosis of HF, still with animals presenting with a normal EF. Furthermore, the group probed the endothelium for molecular features of impaired function including eNOS and Akt phosphorylation.

How is it that the same metabolic (HFD) and cardiovascular stress (L-NAME) model, that is reported equivalent in its implementation, produces different phenotypic outcomes?

One key difference between the study of Srinivas et al. and those published by the laboratory of Joseph Hill is the sub-strain of mouse utilized. The former utilized 8-week old C57BL/6J female mice from Jackson Laboratory, whereas Schiaterella et al. chose 8-week old C57BL/6N mice purchased from Charles River.

Recent developments in genomics along with an increased awareness for experimental reproducibility and rigor, has resulted in a number of reports of the genetic and phenotypic differences between these two sub-strains of black mouse. These two strains originate from one common inbred line established at the Jackson Laboratory in 1948 [11]. A recent genetic analysis reported >500,000 unique coding and non-coding variants distinguishing the two sub-strains of mouse, with phenotypic divergence including in inflammatory response, energy expenditure, glucose tolerance, kidney function and mitochondrial metabolism. Of relevance to cardiovascular physiology, the BL6J strain has been shown to have higher baseline systolic arterial pressure than in N mice, and heart weight to tibia length is significantly lower in BL6N than in BL6J mice. Many of the distinguishing metabolic parameters between BL6J and N have previously been attributed to loss of function mutations in the nicotinamide nucleotide transhydrogenase (*Nnt*) gene BL6J mice, but more recent functional and genomic studies contradicting a monogenic role of *Nnt* as underlying phenotypic differences [12]. The difference in sub-strain of mouse employed in these two studies may explain the non-equivalent response to the same high fat diet and pressure overload induced by L-NAME. Furthermore, even the same mouse sub-strain purchased from a different vendor has demonstrated differential responses to high far feeding [13]. These seemingly similar studies in the field of experimental cardiology reporting different outcomes emphasizes the importance of thorough reporting of methodological approaches and materials. It also remains unknown as to how young male C57BL/6 J mice respond to a HFD and L-NAME (Fig. 1).

**Fig. 1:**
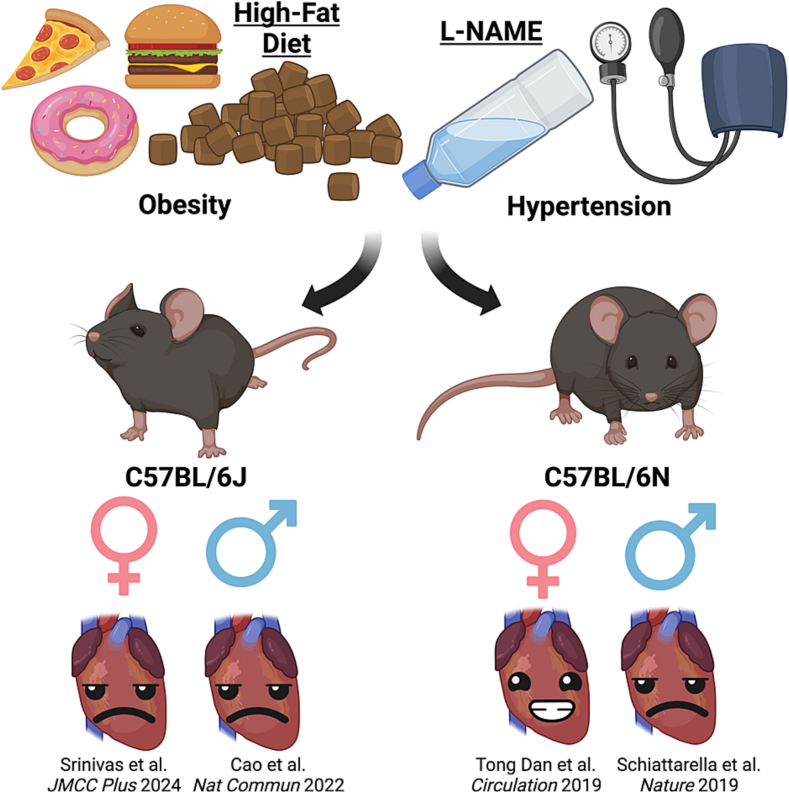
Graphical Abstract: Within 15 weeks of high fat diet feeding (60 % calories from fat) and N(ω)-nitro-L-arginine methyl ester (L-NAME; 0.5gL/) administration in C57BL/6N male (Schiattarella et al.*, Nature* 2019) and in female (Srinivas et al.*, JMCC Plus* 2024) and male (Cao et al.*, Nat Commun* 2022) C57BL/6J mice, diastolic function is compromised, ejection fraction remains normal and pathophysiological signs and symptoms develop. However, the same pre-clinical model in 8-week old C57BL/BN female mice does not recapitulate the model of heart failure with preserved ejection fraction (Tong et al.*, Circulation* 2019).

The present study by Srinivas and colleagues is also not the first to evaluate the sexual dimorphisms in this murine model of diastolic dysfunction and HFpEF. In 2022, Cao et al. revealed both sex- and strain-dependent differences in genes encoding mitochondrial proteins of the myocardium, and described a phenomenon whereby mitochondrial gene expression was significantly correlated with the extent of diastolic dysfunction [14]. Ultimately, this discovery led the authors to ask whether these sexually divergent findings were consistent in the same “two-hit” mouse model of HFpEF. Remarkably, overall mitochondrial function was found to differ substantially between sexes and was also strongly associated with several characteristics of HFpEF, similarly suggesting the existence of an important sex bias in diastolic function.

Whilst the HFD and L-NAME ‘two-hit’ model does effectively recapitulate progressive comorbidity-driven diastolic dysfunction with signs of HF, some elements of human disease are missing from this model. In particular, that of advanced age. HFpEF is a disease of the elderly, predominantly restricted to those aged 60 years or older. Normal aging is accompanied by endothelial dysfunction, LV hypertrophy, altered inflammatory response and wholesale metabolic remodeling. One such pre-clinical model representing HFpEF in an aged small animal is that published by the group of Rudolf de Boer, which combines 12 weeks of HFD feeding and a 4-week low dose Angiotensin II infusion in 18–22 week old female mice [15]. Combining more advanced age, obesity and L-NAME induced pressure overload in experimental animals may prove an even more pathophysiological-relevant and tractable model of this complex cardiometabolic syndrome.

## Funding sources

JT is supported by a K99 Pathway to Independence Award from the 10.13039/100000050NHLBI (1 K99HL166708 - 01A1). ELR acknowledges support from the 10.13039/100000062NIDDK-funded Colorado Nutrition and Obesity Research Center (NORC; P30 DK048520).

## Large language model statement

This article was prepared in the absence of assistance from any LLM or LLM-like program.

## CRediT authorship contribution statement

**Joshua Travers:** Writing – review & editing. **Emma L. Robinson:** Writing – review & editing, Writing – original draft, Conceptualization.

## Declaration of competing interest

The authors declare the following financial interests/personal relationships which may be considered as potential competing interests: JOSHUA TRAVER reports financial support was provided by University of Colorado Anschutz Medical Campus School of Medicine. EMMA L ROBINSON reports financial support was provided by University of Colorado Anschutz Medical Campus School of Medicine. EMMA L ROBINSON reports a relationship with Cell Press that includes: consulting or advisory. If there are other authors, they declare that they have no known competing financial interests or personal relationships that could have appeared to influence the work reported in this paper.
